# Developmentally regulated expression and complex processing of barley pri-microRNAs

**DOI:** 10.1186/1471-2164-14-34

**Published:** 2013-01-16

**Authors:** Katarzyna Kruszka, Andrzej Pacak, Aleksandra Swida-Barteczka, Agnieszka K Stefaniak, Elzbieta Kaja, Izabela Sierocka, Wojciech Karlowski, Artur Jarmolowski, Zofia Szweykowska-Kulinska

**Affiliations:** 1 Department of Gene Expression, Institute of Molecular Biology and Biotechnology, Faculty of Biology, Adam Mickiewicz University in Poznan, Umultowska 89, 61-614, Poznan, Poland; 2 Computational Genomics Laboratory - Bioinformatics Laboratory, Institute of Molecular Biology and Biotechnology, Faculty of Biology, Adam Mickiewicz University in Poznan, Umultowska 89, 61-614, Poznan, Poland

**Keywords:** MicroRNA, Pri-microRNA processing, MicroRNA genes, Splicing, Alternative splicing, Introns, Barley

## Abstract

**Background:**

MicroRNAs (miRNAs) regulate gene expression via mRNA cleavage or translation inhibition. In spite of barley being a cereal of great economic importance, very little data is available concerning its miRNA biogenesis. There are 69 barley miRNA and 67 pre-miRNA sequences available in the miRBase (release 19). However, no barley pri-miRNA and *MIR* gene structures have been shown experimentally. In the present paper, we examine the biogenesis of selected barley miRNAs and the developmental regulation of their pri-miRNA processing to learn more about miRNA maturation in barely.

**Results:**

To investigate the organization of barley microRNA genes, nine microRNAs - 156g, 159b, 166n, 168a-5p/168a-3p, 171e, 397b-3p, 1120, and 1126 - were selected. Two of the studied miRNAs originate from one *MIR168a-5p/168a-3p* gene. The presence of all miRNAs was confirmed using a Northern blot approach. The miRNAs are encoded by genes with diverse organizations, representing mostly independent transcription units with or without introns. The intron-containing miRNA transcripts undergo complex splicing events to generate various spliced isoforms. We identified miRNAs that were encoded within introns of the noncoding genes *MIR156g* and *MIR1126*. Interestingly, the intron that encodes miR156g is spliced less efficiently than the intron encoding miR1126 from their specific precursors. miR397b-3p was detected in barley as a most probable functional miRNA, in contrast to rice where it has been identified as a complementary partner miRNA*. In the case of miR168a-5p/168a-3p, we found the generation of stable, mature molecules from both pre-miRNA arms, confirming evolutionary conservation of the stability of both species, as shown in rice and maize. We suggest that miR1120, located within the 3^′^ UTR of a protein-coding gene and described as a functional miRNA in wheat, may represent a siRNA generated from a mariner-like transposable element.

**Conclusions:**

Seven of the eight barley miRNA genes characterized in this study contain introns with their respective transcripts undergoing developmentally specific processing events prior to the dicing out of pre-miRNA species from their pri-miRNA precursors. The observed tendency to maintain the intron encoding miR156g within the transcript, and preferences in splicing the miR1126-harboring intron, may suggest the existence of specific regulation of the levels of intron-derived miRNAs in barley.

## Background

MicroRNAs are small, single-stranded RNAs, usually 21 nucleotides in length, for the first time identified in *Ceanorhabditis elegans*, and then in various other eukaryotic species which play a key regulatory role in gene expression at the posttranscriptional level [1]. *Arabidopsis thaliana* was the first plant species in which the existence of miRNAs was demonstrated [2-4]. Further studies have confirmed the existence of miRNAs in all plant species studied [5]. The regulatory roles of miRNAs have been demonstrated in plant development, signal transduction, protein degradation, response to environmental stress, and pathogen invasion [6]. Additionally, miRNAs can regulate their own biogenesis, as shown in the case of miR838. The miR838 precursor is localized in the *DICER-LIKE 1* (*DCL1*) intron 14. Dicing out of the pre-miR838 leads to *DCL1* mRNA degradation, which decreases the level of DCL1, a key protein in miRNA biogenesis [7].

miRNAs, together with their almost perfect complementary partners, called miRNAs*, form a duplex located in the stem of a hairpin structure (pre-miRNA). Pre-miRNAs are embedded within primary-miRNAs (pri-miRNAs), which are long products of RNA polymerase II activity that possess their characteristic 5^′^ cap and 3^′^ polyA tail [8]. miRNAs can be located in either arm of a pre-miRNA stem. In plants, the enzyme engaged in trimming the pri-miRNA hairpins, as well as dicing out miRNA-miRNA* duplexes, is called DCL1 [9,10]. The DCL1 together with SERRATE (SE) and HYPONASTIC LEAVES 1 (HYL1), forms microprocessing complex [11,12] that ensures efficiency and accuracy of pri-miRNA to miRNA processing [13]. The efficiency of pri-miRNA recruitment to DCL1-HYL1-SE complex is stimulated by a RNA binding protein TOUGH (TGH) [14], whereas the accuracy of pri-miRNA processing requires HYL1 dephosphorylation triggered by a C-TERMINAL DOMAIN PHOSPHATASE-LIKE 1 (CPL1) [15]. SE cooperates with a cap-binding complex (CBC) to ensure the proper processing of pri-miRNAs [16,17]. Another protein involved in a proper plant miRNA biogenesis is called SICLE (SIC). However, its exact function is elusive [18]. The 3^′^ termini of miRNA/miRNA* duplexes are 2^′^-O-methylated by HUA ENHANCER 1 (HEN1) methyltransferase to prevent 3^′^–5^′^ degradation or 3^′^ uridylation [19-22]. After HASTY (HST)-driven export of the duplex to the cytoplasm, the miRNA is loaded into the RNA-induced silencing complex (RISC) [23,24], while the miRNA* is usually degraded [25]. The miRNA-loaded RISC directs posttranscriptional silencing of the target mRNA, or triggers microRNA-directed phasing during trans-acting siRNA biogenesis [26]. Due to the almost perfect complementarity of miRNA to its target mRNA, it is widely assumed the target mRNA is predominantly cleaved [27,28]. However, there are examples of translation suppression without mRNA cleavage, as has been shown for the ath-miR172-triggered downregulation of APETALA2 expression level [29]. Similar to animal models, studies on *A. thaliana* miRNA-action mutants have revealed the existence of translational repression by miRNAs [30].

As previously mentioned, miRNA biogenesis produces obligatory side products from DCL1-triggered cleavage of pre-miRNAs that results in miRNAs* originating from the strand opposite to the mature miRNA. While the expression of miRNAs* is rarely detected due to their rapid degradation [25], there are some examples of animal miRNA* that remain stable. It has been shown that these stable miRNA* are incorporated into RISC complexes to posttranscriptionally downregulate mRNA translation [31]. It has been demonstrated in the case of human miRNA-155 and its partner miRNA-155*, that both of them regulate type I interferon production [32]. An increasing amount of deep sequencing data for plant small RNAs has provided a basis for describing some examples of substantial miRNA* representation. The physiological roles of these molecules have yet to be established. Supposing miRNAs* participate in the posttranscriptional silencing of targeted mRNAs, it is still not known whether they are involved in regulation of the same biological pathway as their cognate miRNAs. Nevertheless, it has been demonstrated for the miR393/miR393* pair that they both regulate plant immune responses through different cellular pathways [33].

Although hundreds of plant miRNAs have been identified, their pri-miRNAs and genes are mostly unknown. As was demonstrated for *Arabidopsis*, plant miRNA precursors can be hundreds or thousands of nucleotides long, with many containing one or more introns that undergo constitutive or alternative splicing [34]. While plant *MIR* genes are predominately located in intergenic loci, there have been an increasing number of examples of intragenic *MIR* loci located within introns of protein coding genes [7,35,36]. The mirtrons represent a class of intron-encoded miRNAs which are processed from spliced-out introns and constitute hairpin substrates for the dicing machinery [37-39]. There are 24 mirtrons identified in different plant species: five in *A. thaliana*, 18 in rice, and one in cassava (*Manihot esculenta*) [38,40,41]. The existence of plant polycistronic miRNA genes that carry multiple miRNAs has been shown in *A. thaliana, Oryza sativa* and *Physcomitrella patens*[42-44]. Bioinformatic analyses suggest that *MIR* genes may also overlap with protein coding genes. In *A. thaliana*, the *MIR777* gene partly covers the 5^′^ UTR of the protein-coding gene *At1g70650*[7]. Another surprising finding was the discovery of osa-miR3981, which is located in the last exon of the putative glyoxylase mRNA in rice [45]. These reports suggest there might be additional examples of *MIR* genes overlapping with protein-coding genes. Regulated co-expression of protein/miRNA-coding genes remains unknown.

Barley is an economically important monocotyledonous crop plant. However, little is known about barley miRNA precursors and there is no data on *MIR* gene structures. High-throughput sequencing of conserved and novel small RNAs is widely used for computational scanning of genomic sequences in search of miRNA genes. A bioinformatic search of EST sequences resulted in a set of predicted potential pre-miRNAs, but until now there has been no experimental evidence validating these data [46,47]. Currently, deep sequencing of barley small RNAs gained a set of detailed information concerning mature barley miRNAs [48,49]. Lv et al. [49] has identified 259 mature miRNAs (126 conserved and 133 novel miRNAs) from which 46 miRNAs were deposited into miRBase (realease 19, http://www.mirbase.org/index.shtml) [50,51]. Currently, there are 69 barley miRNA sequences and 67 pre-miRNA structures deposited in miRBase. However, none of the precursor structures have been validated using experimental approaches. Moreover, primary miRNA transcripts, as well as the genes for barley miRNAs, remain unknown.

In this study, we show experimental evidence for nine conserved barley miRNAs and their pre- and pri-miRNA precursors, as well as their gene structures. Our findings reveal a diverse organization of barley *MIRNA* genes, as well as expression levels of pre-miRNA and miRNA primary transcripts in five barley developmental stages studied. Furthermore, we show complex processing events of pri-miRNAs, with different pathways leading to various levels of mature miRNAs being observed in particular developmental stages. A regulatory role of posttranscriptional processing of pri-miRNAs is postulated.

## Results and discussion

To determine the structures of miRNA genes and their transcripts, we selected eight barley cDNA nucleotide sequences for microRNAs precursors - 156g, 159b, 166n, 168a-5p/168a-3p, 171e, 397b-3p, 1120 and 1126 - deposited in GeneBank, http://www.ncbi.nlm.nih.gov/[52] (see Methods) for which computationally predicted hairpin structures, carrying conserved or newly estimated miRNA homologues, were described [46,47]. Moreover, we followed their expression profiles at the level of pri-miRNAs, pre-miRNAs, and mature miRNAs in the following developmental stages: 1-, 2-, 3-, and 6-week-old, and 68-day-old plants. We designed 5^′^ and 3^′^ RACE primers to determine the full-length sequences of the pri-miRNA transcripts for all pri-miRNAs analyzed. On the basis of the nucleotide sequences and hairpin structural similarities, we have identified the barley *MIRNA* genes in this study as the orthologues of corresponding genes in rice or wheat. We named the barley *MIRNA* genes according to their matching rice or wheat orthologues. The total length of the pri-miRNA precursors was calculated on the basis of the longest 5^′^ and 3^′^ RACE products. For all barley pri-miRNAs, RT-PCR was carried out using primers designed against the 5^′^ and 3^′^ ends of the longest pri-miRNA RACE products to confirm that the longest pri-miRNA 5^′^ and 3^′^ ends belong to the same precursor molecule. In addition, qRT-PCR was performed to compare the level of all pri-miRNAs in the developmental stages studied.

We designed 5^′^ and 3^′^ genome walking primers to determine the sequences and structures of the barley *MIRNA* genes. The intron-exon structure of the barley *MIRNA* genes was established by alignment of the pri-miRNA sequences coming from RACE and RT-PCR experiments with genomic fragments obtained by genome walking, as well as by using the FSPLICE program [53]. The structure and length for each barley *MIRNA* gene, the position of its miRNA within the gene, and its rice or wheat orthologues are presented in Table 1.

**Table 1:** **The lengths and structures of eight characterized *H. vulgare MIRNA *genes**

**No.**	***H. vulgare MIRNA *gene**	**Length [bp]**	Position of mature miRNA and miRNA* sequences within gene^**#**^	**Number of exons [length in bp]**	**Number of introns [length in bp]**	**Rice (osa)/wheat (tae) orthologues^**
1.	156g	9759	255-275 (intron 1) 349-370*	6 [126, 116, 68, 83, 145, 513]	5 [851, 1024, 4666, 76, 2091]	osa-miR156g
2.	159b	1251	392-412 (exon 1) 241-261*	2 [596, 249]	1 [406]	osa-miR159b
3.	166n	1237	359-379 (exon 1) 304-324*	2 [510, 469]	1 [258]	osa-miR166n
4.	168a-5p/168a-3p	1004	133-153 (exon 1) 180-200*	2 [261, 502]	1 [241]	osa-miR168a
5.	171e	1095	150-170 (exon 1) 95-115*	2 [296, 486]	1 [313]	osa-miR171e
6.	397b-3p	868	588-608 (exon 1) 520-540*	1 [868]	0	osa-miR397b
7.	1120	3606	3288-3311 (exon 7) 3238-3260*	7 [97, 163, 304, 201, 144, 179, 697]	6 [111, 284, 275, 151, 94, 906]	tae-miR1120
8.	1126	3297	1242-1264 (intron 3) 1309-1331*	7 [65, 74, 40, 190, 218, 76, 120]	6 [150, 203, 1305, 91, 605, 160]	tae-miR1126

^#^ The gene nucleotide numeration starts at the first nucleotide of the gene.

^ Based on nucleotide sequence and hairpin structure similarities we classified barley *MIRs* as orthologues of rice or wheat.

### miR397b-3p: miR* or functional miRNA?

**Figure 1:**
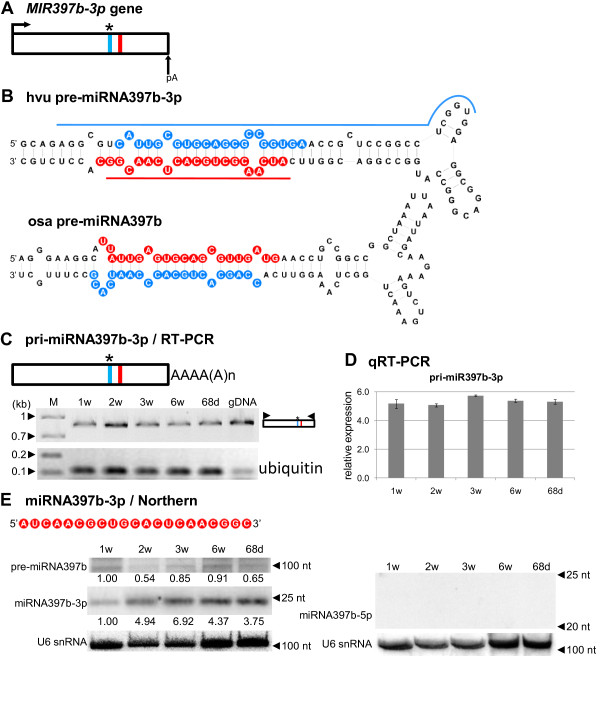
**Schematic representation of the *MIR397b-3p *gene and its precursors. Detection of pri-, pre- and mature miR397b-3p.** (**A**) *MIR397b-3p* gene structure; left arrow indicates putative transcription start site; arrow marked as pA depicts precursor polyadenylation site. (**B**) pre-miRNA397b-3p hairpin structure (ΔG=−70.8 kcal/mol) and its rice orthologue (ΔG=−51.2 kcal/mol); the blue line indicates the region of the pre-miRNA from which the hybridization probe for precursor detection was designed, while the red line highlights the probe for detection of the mature miRNA. (**C**) Structure of pri-miRNA397b-3p (upper panel); RT-PCR analysis of its expression in five barley developmental stages (lower panel); primer positions are marked by black triangles on the pri-miRNA graph. (**D**) Real-time PCR measurements of pri-miRNA397b-3p expression level; bars on a chart represent standard deviation. Values are shown as the mean ± SD (n=3) from three independent experiments. (**E**) Nucleotide sequence of the mature miRNA397b-3p molecule; detection of pre-miRNA (left upper panel), mature miR397b-3p (left middle panel), and miR397b-5p (right panel) using Northern hybridization. U6 was used as a loading control. The level of pre-miRNA and miRNA in 1-week-old plants was arbitrarily assumed to be ‘1’, and the levels of pre-miRNA and miRNA were quantified relative to this at all other developmental stages. The miRNA is marked in red, the miRNA* in blue; 1w: one-week-old seedlings, 2w: two-week-old seedlings, 3w: three-week-old plants, 6w: six-week-old plants, 68d: 68-day-old plants, gDNA: genomic DNA; M - GeneRuler 100 bp Plus or 1kb Plus DNA Ladders.

Surprisingly, out of the eight *MIR* genes analyzed, *MIR397b-3p* was the only intronless gene (Figure 1A, Table 1). Based on the nucleotide sequence and hairpin structural similarities, we classified barley *MIR397* as an orthologue of rice *MIR397b* (Figure 1)B. The annotated osa-miR397b is located in the 5^′^ arm of the hairpin structure. We failed to detect barley miR397b-5p molecules corresponding to osa-miR397b when Northern hybridization was used (Figure 1E, right panel). Surprisingly, we detected relatively high levels of barley miR397b-3p, the molecule that corresponds to rice miR397b* (Figure 1E, left middle panel). This result may suggest that miR397b-3p is a functional microRNA molecule in *Hordeum vulgare.* This observation is in agreement with the recently published results of Jeong et al. [54], who showed that in rice, relatively highly expressed annotated miRNA*s of miR529a, miR1430, and miR1433 are likely to be true miRNAs and not miRNA*s.

Detailed analysis showed that the lowest level of miR397b-3p was observed in 1-week-old plants, while the highest level was seen in 3-week-old plants (Figure 1E, left middle panel). Using a hybridization probe complementary to the 5^′^ arm of the stem and loop of the hairpin structure, we were able to detect the precursor of miR397b-3p, which is approximately 110 nucleotides (nt) long. Amplification of the whole pri-miR397b-3p (Figure 1C) as well as real-time PCR experiments (Figure 1D) revealed the presence of a single transcript expressed almost equally in all developmental stages examined. However, there was no correlation between the level of precursor and mature miR397b-3p, most likely due to detection of putative miR397 molecules belonging to the same microRNA397 family encoded by other loci.

### pre-miR159b belongs to the longest known barley precursors’ hairpins

**Figure 2:**
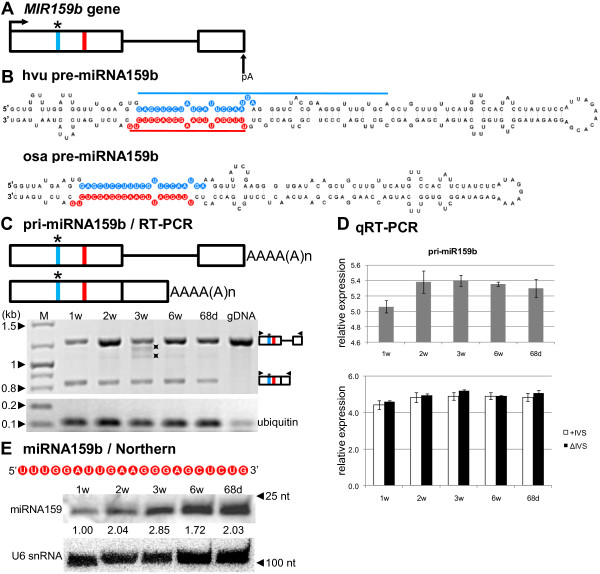
**Schematic representation of the *MIR159b *gene and its precursors. Detection of pri- and mature miR159b.** (**A**) *MIR159b* gene structure. (**B**) pre-miRNA159b hairpin structure (ΔG=−95 kcal/mol) and its rice orthologue (ΔG=−79.3 kcal/mol); blue and red lines indicate hybridization regions as described in Figure 1 (**C**) pri-miRNA159b structures (upper panel) and RT-PCR analysis of their expression in five barley developmental stages studied (lower panel). (**D**) Real-time PCR measurements of total pri-miRNA159b expression level (upper graph) and its spliced (ΔIVS) and unspliced variants (+IVS) (lower graph); bars on the charts represent standard deviation. Values are shown as the mean ±SD (n=3) from three independent experiments. (**E**) Nucleotide sequence of the mature miR159b molecule, and detection of mature miR159b using Northern hybridization. U6 was used as a loading control. The levels of pre-miRNAs and miRNA were calculated as described in Figure 1. Colors, abbreviations, and symbols as in Figure 1; asterisks next to bands on agarose gel mark nonspecific products.

The detailed structures of the *MIR159b* gene and its pre-miRNA are shown in Figure 2A and B. Comparison of the barley pre-miR159b stem and loop structure to 67 barley pre-miRNAs deposited in miRBase (release 19) revealed that this precursor forms one of the longest hairpins which is 224 nt long (Figure 2)B. RT-PCR of full-length barley pri-miR159b transcripts demonstrated the presence of both unspliced (+IVS, **I**nter**V**ening **S**equence) and spliced isoforms (ΔIVS) (Figure 2)C with almost the same expression level (Figure 2D, lower graph). We observed high expression levels of mature miR159 using Northern hybridization, however, we failed to detect pre-miR159b, which was most likely a result of rapid processing of the pre-miRNA (Figure 2E). Our observations show that the level of mature miR159 fluctuates in various developmental stages with the lowest expression level detected in 1-week-old plants, which is in agreement with the real-time PCR results for pri-miR159b (Figure 2D, upper graph). It has to be noted that Northern analysis shows the expression of all putative mature miR159 family members. In *Arabidopsis,* three members of the miR159 family were annotated - miR159a, b and c [3,55] - while for monocotyledonous species such as rice and maize, eight and fourteen members of the miR159 family have been described, respectively [4,43,56,57]. Lv et al. [49] described three barley miR159 species with different nucleotide sequences. miR159b described by us differs in its nucleotide sequence from the three species found by Lv et al. [49]. Our studies, together with recent results of Lv et al. and miRBase, reveal that the miR159 family in barley consists of at least four members.

### *MIR166n* generates two transcripts with heterogeneous, developmentally specific 5′ ends

**Figure 3:**
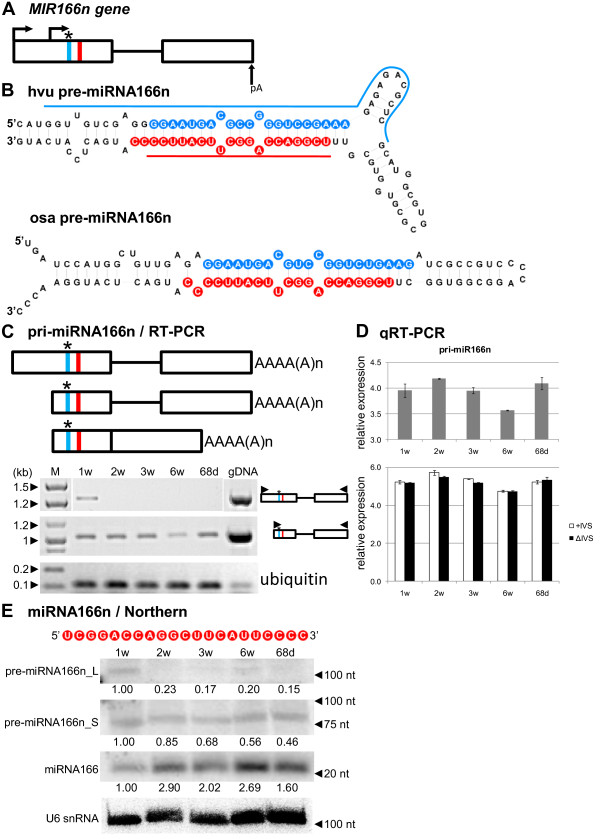
**Schematic representation of the *MIR166n *gene and its precursors. Detection of pri-, pre- and mature miR166n.** (**A**) *MIR166n* gene structure. (**B**) pre-miRNA166n hairpin structure (ΔG=−61 kcal/mol) and its rice orthologue (ΔG=−52.3 kcal/mol); blue and red lines indicate hybridization regions as described in Figure 1. (**C**) pri-miRNA166n structures (upper panel); RT-PCR analysis of their expression in five barley developmental stages studied (lower panel). (**D**) Real-time PCR measurements of total pri-miRNA166n expression level (upper graph) and its spliced (ΔIVS) and unspliced variants (+IVS) (lower graph); bars on the charts represent standard deviation. Values are shown as the mean ±SD (n=3) from three independent experiments. (**E**) Nucleotide sequence of the mature miR166n molecule, and detection of pre-miRNA166n long (L) and short (S) intermediates, and mature miR166n using Northern hybridization. U6 was used as a loading control. The levels of pre-miRNAs and miRNA were calculated as described in Figure 1. Colors, abbreviations, and symbols as in Figure 1.

The detailed structure of the *MIR166n* gene and its pre-miRNA are shown in Figure 3A and B. miR166 is represented by a very large gene family in both monocot and dicot plants. Its members target mRNAs coding for *HD-ZIPIII* transcription factors, including *Phabulosa (PHB)* and *Phavoluta (PHV)*, which regulate axillary meristem initiation and leaf development [58,59]. In *Arabidopsis,* the miR166 family consists of seven members [3,8], whereas in both rice and maize, 14 miR166s were annotated [3,4,57,60,61]. Concerning the barley miR166 family, so far only three members, including miR166a, b and c, have been annotated in miRBase (release 19) among which only miR166b has been experimentally confirmed [48]. However, despite the identical sequences of mature miR166b and miR166n species, the nucleotide sequences of their pre-miRNAs are different, confirming that the miR166n described in this paper is a novel member of the miR166 family in barley. Interestingly, we identified two heterogeneous 5^′^ ends for the *MIR166n* transcript, separated by 216 nt (Figure 3C). Using 5^′^ and 3^′^ flanking primers for pri-miR166n amplification, we detected two transcript variants: a longer isoform present only in 1-week-old plants, and a shorter form detected in all developmental stages analyzed (Figure 3C). In the case of the 1-week-old plants, it is possible that we amplified the shorter transcript using the longer one as a template. However, it was not possible to amplify the longer transcript using the shorter one as a template in the case of 2-, 3-, 6-week and 68-day-old plants. This heterogeneity in the 5^′^ ends of pri-miRNA was also found in *Arabidopsis* miRNA precursors. For example, pri-miR169f is represented by two transcripts with 5^′^ ends that are 3 nt apart, while for pri-miR172e, the two most distal of the three identified 5^′^ ends are 214 nt apart [34].

The qRT-PCR analysis confirmed the presence of the miR166n precursor in all developmental stages tested (Figure 3D, upper graph). The lowest expression level of the pri-miR166n was detected in 6-week-old plants as it was also detected in RT-PCR experiments (Figure 3C). In addition, we analyzed the existence of spliced (ΔIVS) and unspliced (+IVS) isoforms of the miR166n precursor (Figure 3D, lower graph). During barley development, both the spliced and unspliced precursor variants were present on an almost equal level in each growth stage tested.

Using Northern hybridization, we identified two pre-miR166n forms varying in length by approximately 30 nt (Figure 3E). The shorter form (pre-miR166n_S) was around 75 nt long, while the other (pre-miR166n_L) was about 100 nt long. The shorter form of the miR166n precursor may represent the hairpin structure, with the stem having miRNA and miRNA* at the base of this hairpin, while the longer form may correspond to the same hairpin form with an extended stem. The shorter form of pre-mR166n is more abundant compared to the longer one. The highest expression level of pre-miR166n detected in 1-week-old plants corresponded to the lowest level of mature miR166 observed in the same growth stage. The level of the mature miR166 reached its highest level in 2-week-old plants. In general, our observations show that the miR166 level fluctuates when various developmental stages are compared (Figure 3E).

### miR168a-5p and miR168a-3p are generated from the same precursor and differ in their expression levels

**Figure 4:**
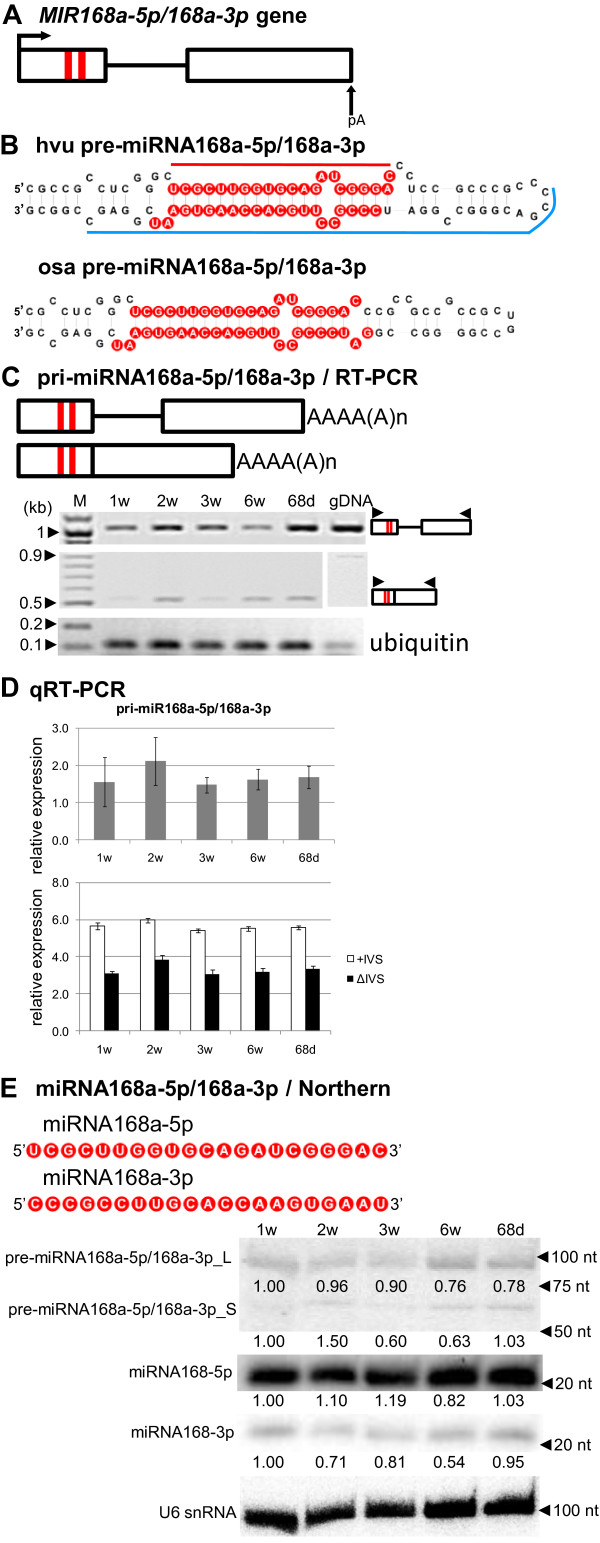
**Schematic representation of the *MIR168a-5p/168-3p *gene and its precursors. Detection of pri-, pre-, and mature miR168-5p and miR168a-3p.** (**A**) *MIR168a-5p/168-3p* gene structure. (**B**) pre-miRNA168a-5p/168-3p hairpin structure (ΔG=−60.7 kcal/mol) and its rice orthologue (ΔG=−52.2 kcal/mol); blue and red lines indicate hybridization regions as described in Figure 1. (**C**) pri-miRNA168a-5p/168-3p structures (upper panel) and RT-PCR analysis of their expression in five barley developmental stages (lower panel). (**D**) Real-time PCR measurements of pri-miRNA miRNA168a-5p/168-3p expression levels (upper graph) and its spliced (ΔIVS) and unspliced variants (+IVS) (lower graph); bars on the charts represent standard deviation. Values are shown as the mean ±SD (n=3) from three independent experiments. (**E**) Nucleotide sequences of the mature miR168a-5p and miR168a-3p molecules, and Northern detection of pre-miRNA168a-5p/168-3p long (L) and short (S) intermediates, mature miR168-5p and miR168a-3p. U6 was used as a loading control. The levels of pre-miRNAs and miRNA were calculated as described in Figure 1. Colors, abbreviations, and symbols as in Figure 1; asterisk next to band on agarose gel marks nonspecific product.

The detailed structures of *MIR168a-5p/168a-3p* gene and its pre-miRNA are shown in Figure 4A and B. miR168 is a relatively small family consisting of only two members in *A. thaliana* (miR168a and miR168b), three members in *O. sativa* (miR168a-5p/miR168a-3p, miR168b) and four in *Zea mays* (miR168a-5p/168a-3p, miR168b5-p/miR168b-3p) [3,4,8,56]. In *Arabidopsis*, miR168 targets mRNA coding for the Argonaute (AGO1) protein, which is crucial for miRNA function; hence miR168 is involved in a negative-feedback mechanism for controlling all miRNAs biogenesis [58,62]. Real-time PCR data have shown that transcripts of *MIR168a-5p/168a-3p* were present in all developmental stages tested (Figure 4C and D, upper graph). The expression level of the intron-containing isoform (+IVS) is higher than of the isoform without intron (ΔIVS) in all developmental stages studied (Figure 4D, lower graph). Similar to pre-miR166n, the expression of two different forms of the miR168a-5p/miR168a-3p precursor was detected by Northern analysis. The shorter form of the precursor (miR168a-5p/miR168a-3p_S), approximately 70 nt long, may correspond to the pre-miRNA having miR168a-5p and miR168a-3p located at the base of the stem, while the longer form (miR168a-5p/miR168a-3p_L) has the extended stem. The expression level of the longer precursor was elevated compared to expression of the shorter one (Figure 4E).

Interestingly, in addition to the expression of mature miR168-5p, we also observed the expression of miR168-3p (Figure 4E). There are two possible explanations for this observation: (i) as postulated for rice and maize, both miR168-5p and miR168-3p molecules could be functional in barley [57,63] or (ii) miR168-3p represents a relatively stable molecule of miRNA*. It should be noted that a target mRNA sequence for the miR168-3p molecule has not yet been identified. Using psRNATarget software (expectation=3.0), we identified a potential target barley mRNA [GeneBank: AK364646.1] encoding a protein similar to a ubiquitin-like protein from *Triticum aestivum*. The expression level of the mature miR168-5p is notably stronger than the miR168-3p. The expression profiles of miRNA168-5p/168-3p in particular developmental stages also differed, which may suggest a functional role of both miRNA species, as was reported for miR319b and miR319b.2 in *Arabidopsis*[64].

### Intron-containing pri-miR171e undergoes complex splicing events during development

**Figure 5:**
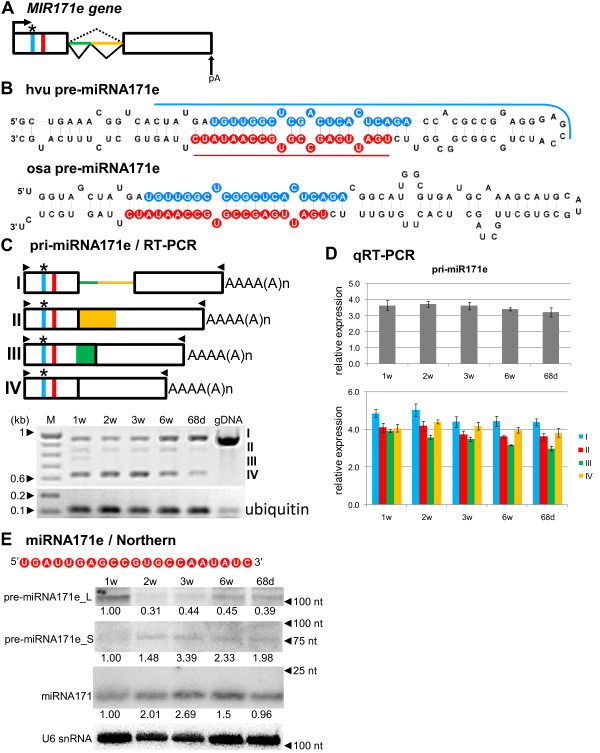
**Schematic representation of the *MIR171e *gene and its precursors. Detection of pri-, pre- and mature miR171e.** (**A**) *MIR171e* gene structure. (**B**) pre-miRNA171e hairpin structure (ΔG=−59.1 kcal/mol) and its rice orthologue (ΔG=−58.9 kcal/mol); blue and red lines indicate hybridization regions as described in Figure 1. (**C**) pri-miRNA171e structures (upper panel), green and yellow colors show alternatively retained transcript fragments as a consequence of alternative splicing events; RT-PCR detection of pri-miRNA171e expression in five barley developmental stages (lower panel). (**D**) Real-time PCR measurements of total pri-miRNA171e expression levels (upper graph) and its splice variants (I–IV) (lower graph); bars on the charts represent standard deviation. Values are shown as the mean ± SD (n=3) from three independent experiments. (**E**) Nucleotide sequence of the mature miR171e molecule, detection of pre-miRNA171e long (L) and short (S) intermediates, and mature miR171e using Northern hybridization. U6 was used as a loading control. The levels of pre-miRNAs and miRNA were calculated as described in Figure 1. Colors, abbreviations, and symbols as in Figure 1.

The detailed structures of *MIR171e* gene and its pre-miRNA are shown in Figure 5A and B. In the case of the miR171e precursor, alternatively spliced transcripts were detected (Figure 5C). What is more interesting the single U2 intron can undergo three independent events of alternative splicing. First, proximal to the 5^′^ end of pri-miRNA, 5^′^ and 3^′^ splice sites (ss) can be selected resulting in extension of the 3^′^ exon by 218 nt (variant II). Second, distal 5^′^ ss and 3^′^ ss can be selected that result in the 5^′^ exon’s length increasing by 95 nt (variant III). Third, proximal 5^′^ ss and distal 3^′^ ss can be selected, resulting in the removal of a 313 nt long intron (variant IV). The long intron sequence (present in the original primary transcript and marked as variant I) covers both shorter introns described above (Figure 5C, upper panel). All four variants were confirmed by RT-PCR and the subsequent sequencing of the amplification products (Figure 5C, lower panel). We cannot rule out the possibility that the fully spliced precursor may also be a product of a two-step splicing event, where proximal 5^′^ ss and proximal 3^′^ ss are selected in the first step, and distal 5^′^ ss and distal 3^′^ ss at the second one. The level of pri-miR171e is almost the same during developmental stages as revealed by qRT-PCR measurements (Figure 5D, upper graph). All four isoforms of the miR171e precursor were detected using real-time PCR (Figure 5D, lower graph). The expression level of the isoform I containing full-length intron was the highest in all developmental stages tested. The lowest expression of the isoform III might explain the difficulties with its RT-PCR detection using the peripheral primers (Figure 5C).

Using Northern hybridization, we detected the mature miR171 in all growth stages tested, with a notably elevated level in 3-week-old plants (Figure 5E). Northern analysis also revealed two pre-miR171e variants - a shorter one of approximately 75 nt (pre-miR171e_S), which might correspond to the precursor of the hairpin structure with miR171e/miR171e* at the base of the stem, and a longer variant, approximately 110 nt long (pre-miR171e_L), which might represent the same precursor with the extended stem. Interestingly, the level of shortened pre-miRNA171e was highest in the 3-week-old plants, which corresponds to the highest level of the mature miRNA observed in the same developmental stage. Taken together, the developmental analysis of the processing of the miR171e transcript shows a complex landscape of various splicing events that suggests potential regulatory role of splicing in mature microRNA biogenesis.

### miR156g and miR1126 are encoded within introns of noncoding genes

**Figure 6:**
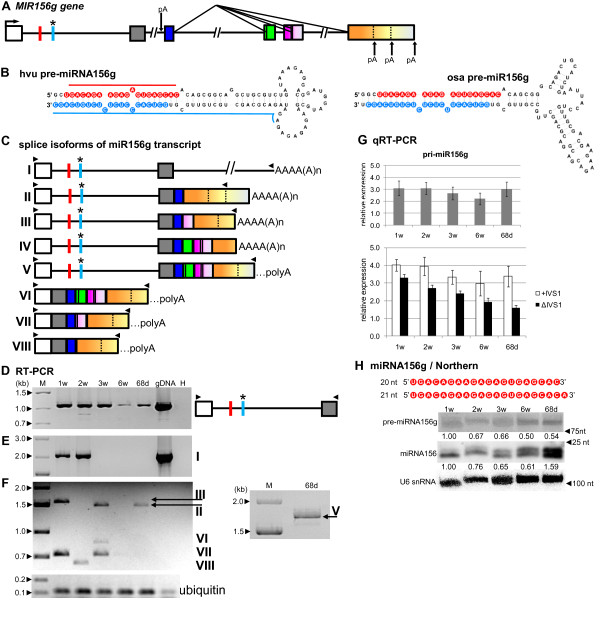
**Schematic representation of the *MIR156g *gene and its precursors. Detection of pri-, pre- and mature miR156g.** (**A**) *MIR156g* gene structure; thin black vertical bars within exons show additional splice sites identified during pri-miRNA156g analyses; dotted-vertical lines within the last exon together with pA symbols denote polyadenylation sites. (**B**) pre-miRNA156g hairpin structure (ΔG=−65.85 kcal/mol) and its rice orthologue (ΔG=−61.2 kcal/mol); blue and red lines indicate hybridization regions as described in Figure 1. (**C**) Structures of splice isoforms (I–VIII) of the miR156g transcript; …polyA indicates a putative polyA site in splice isoforms as the determination of an accurate polyA site for PCR products is not possible. (**D**) RT-PCR analysis of first intron retention throughout barley plant life stages. (**E–F**) pri-miRNA156g RT-PCR expression analysis in five barley developmental stages. Arrows on agarose gel indicate splice isoforms II, III and V. (**G**) Real-time PCR measurements of total pri-miRNA156g expression levels (upper graph) and pri-miR156g fragments carrying the first intron (+IVS1) and after the first intron splicing (ΔIVS1) (lower graph); bars on the charts represent standard deviation. Values are shown as the mean ±SD (n=3) from three independent experiments. (**H**) Nucleotide sequence of the mature miR156g molecule, and detection of pre-miRNA and mature miR156g using Northern hybridization. U6 was used as a loading control. The levels of pre-miRNAs and miRNA were calculated as described in Figure 1. Colors, abbreviations, and symbols as in Figure 1. Additional colors depict alternatively spliced exons in the pri-miRNA.

**Figure 7:**
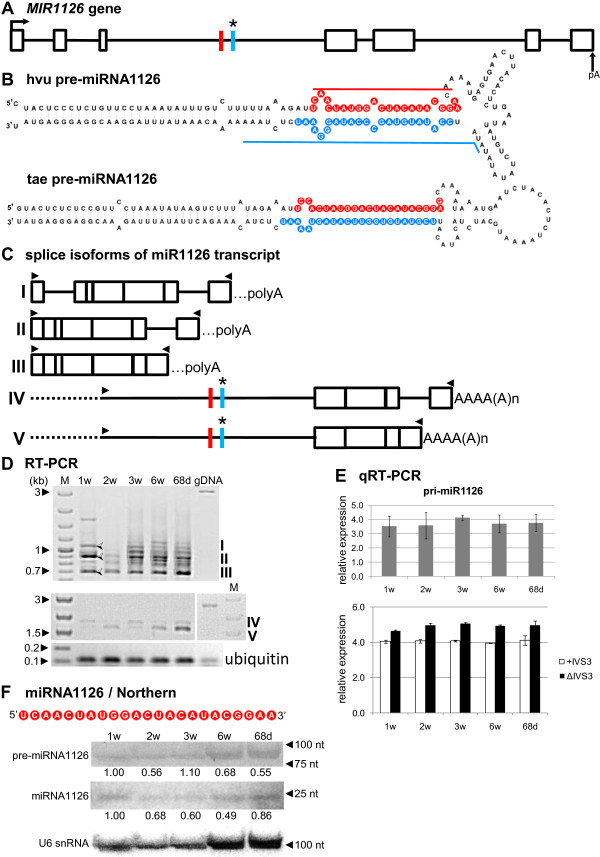
**Schematic representation of the *MIR1126 *gene and its precursors. Detection of pri-, pre- and mature miR1126.** (**A**) *MIR1126* gene structure. (**B**) pre-miRNA1126 hairpin structure (ΔG=−78.4 kcal/mol) and its wheat orthologue (ΔG=−73.27 kcal/mol); blue and red lines indicate hybridization regions as described in Figure 1. (**C**) Structures of splice isoforms (I–V) of the miR1126 transcript; dashed lines represents unamplified 5^′^ fragments of the noncoding RNA isoforms IV and V; …polyA indicates a putative polyA site in splice isoforms as the determination of an accurate polyA site for PCR products is not possible. (**D**) RT-PCR expression analysis of splice isoforms (I–V) of the miR1126 transcript in all barley developmental stages studied. Half-open arrows on agarose gel indicate specific, identified products. (**E**) Real-time PCR measurements of total pri-miRNA1126 expression levels (upper graph) and pri-miR1126 fragments carrying the third intron (+IVS3) and after the third intron splicing (ΔIVS3) (lower graph); bars on the charts represent standard deviation. Values are shown as the mean ±SD (n=3) from three independent experiments. (**F**) Nucleotide sequence of the mature miR1126 molecule, and detection of pre-miRNA and mature miR1126 using Northern hybridization. U6 was used as a loading control. The levels of pre-miRNAs and miRNA were calculated as described in Figure 1. Colors, abbreviations, and symbols as in Figure 1.

The *MIR156g* and *MIR1126* genes are especially interesting, since their precursors were found within introns (intron 1 and 3, respectively). The position of miR156g is close to the 5^′^ end of the first intron, while the miR1126 sequence is located almost in the middle of the third intron (see Table 1, Figure 6A, and Figure 7A). Our analysis has revealed that open reading frames (ORFs) can be identified in both genes. Since they are relatively short (putative 59 amino acids (aa) to putative 150 aa – Additional file 1) without significant similarities to any protein sequences deposited in various protein databases, we consider these genes as noncoding. However, it has been shown that 36 nt and 72 nt ORFs in the 5^′^ end of a legume *ENOD40* gene (encoding one of the earliest nodulins) are translated into two short peptides involved in the control of sucrose use in nitrogen-fixing nodules [65]. The presence of the potential proteins encoded by *MIR156g* and *MIR1126* genes should therefore be verified by further studies.

The *MIR156g* gene consists of six exons and five introns with both miR156g and miR156g* sequences localized within the first intron (Table 1, Figure 6A). All introns carry U2-type signatures. Based on nucleotide sequence and structural similarities, we classify barley *MIR156* as an orthologue of rice *MIR156g* (Figure 6B). Our analysis revealed that miR156g from rice is also intron-encoded, within the P0701F11.20 gene, described as encoding a hypothetical 132 aa protein. This finding suggests an evolutionary conservation of intron-encoded miR156g organization.

Using 5^′^ and 3^′^ RACE results and full transcript analyses, we detected eight splice isoforms (I–VIII, Figure 6C). Isoform I is polyadenylated within the second intron, while isoforms II to VIII are polyadenylated within the last exon (Figure 6A and C). Isoforms I–V maintain the first intron in which miR156g is embedded, but the remaining bodies of the precursors represent various alternatively spliced variants. Isoforms VI–VIII do not contain the microRNA-encoding intron, and while still representing splice variants of the same gene transcript, they cannot be named as genuine miRNA precursors. This high number of splice variants of the *MIR156g* gene indicates a very complex processing of its transcript.

The level of pri-miR156g is the lowest in the 6-week-old plants as revealed by qRT-PCR analysis (Figure 6G, upper graph). Using primers anchored in exons 1 and 2, we confirmed the presence of the unspliced isoform containing pre-miR156g in all developmental stages (Figure 6D). Real-time PCR analyses revealed that the expression level of the transcript with spliced intron 1 was gradually decreasing, while the level of the intron 1 containing product fluctuates during development (Figure 6G, lower graph).

Using primers anchored in exon 1 and the second intron, we detected RT-PCR products containing intron 1 in 1- and 2-week-old plants, while there was no product in the other three developmental stages (Figure 6E). These results suggest that intron 2 is spliced from the precursor more efficiently than intron 1 (see Figure 6D). Using primers specific for the first and last exons, we amplified various RT-PCR products of partially spliced *MIR156g* transcripts (Figure 6F). The obtained pattern of RT-PCR splice isoform products is very complex because of dynamic processing events. Therefore the observed complex pattern of multiple splice isoforms can slightly vary between biological replicates. The introns numbered 2–5 were spliced in all identified products. The only intron maintained was the first one containing pre-miR156g. These results confirm and extend our previous suggestion that intron 1 is spliced less efficiently, not only in comparison to the second intron, but also compared to the other introns of pri-miR156g.

Northern hybridization confirmed expression of the approximately 120nt long pre-miR156g in all growth stages tested. Hybridization also revealed the presence of two mature miR156, 20 and 21 nt long (Figure 6H). The 20 nt long mature miR156 was previously identified in barley using deep sequencing [48]. A 21 nt long mature miR156 with an additional adenosine residue at the 3^′^ end is annotated in the databases of many eukaryotic species [50,51]. Both the 20 and 21 nt miR156 species were equally represented in 6-week- and 68-day-old plants; however, in 1- and 2-week-old plants, primarily the 20 nt long miR156 was detectable. Both 20 and 21 nt long miR156 were expressed at the highest level in 68-day-old plants.

The *MIR1126* gene consists of seven exons and six introns, with miR1126 and miR1126* localized within the third intron (Table 1, Figure 7A and B). All introns are U2 type. A search for the presence of ORF regions within the gene sequence resulted in the identification of three putative sequences: 80 aa, 87 aa, and the longest 91 aa (see Additional file 1). No conserved domains were detected in these coding sequences (CDS) and no significant similarities to known proteins were found. Thus, we concluded that the identified ORFs most probably do not represent CDS and do not encode proteins.

In the case of the intron-encoded miR1126 precursor, similar to pri-miR156g, a plethora of splicing isoforms was observed when 5^′^ and 3' RACE experiments, as well as RT-PCR amplifications of the full transcripts, were carried out. Among the many RT-PCR products, we identified five splice isoforms (I–V, Figure 7C and D). We found the fully spliced transcript (splice isoform III), as well as transcripts retaining the last intron (splice isoform II), and an isoform retaining both the first and the last introns (splice isoform I) (Figure 7C and D, upper panel). In contrast to *MIR156g*, none of the identified spliced isoforms contained miR1126 or miR1126*, thus they cannot be named pri-miRNAs. Using a 5^′^ primer anchored in the third intron upstream of miR1126/miR1126*, and the most peripheral 3^′^ primer anchored in the last exon, we were able to amplify precursors containing the intron sequence with miR1126 and miR1126* in all developmental stages studied (Figure 7D, middle panel). Real-time PCR analyses confirmed that splice isoforms I–III lacking intron 3 were present in higher amount in comparison to the precursor isoforms IV and V containing intron 3 (Figure 7E, lower graph). The observed tendency to maintain the intron-containing miR156g within the transcript, and preferences in splicing the miR1126-harboring intron, may suggest the existence of special regulation of the levels of intron-derived miRNAs in barley.

qRT-PCR of pri-miR1126 shows the highest expression level in 3-week-old plants (Figure 7E, upper graph), which is in agreement with the highest level of pri-miR1126 in 3-week-old plants detected by Northern hybridization (Figure 7F). The mature barley miR1126 molecule is 23 nt long, the same as reported for wheat miR1126 (Figure 7F). A sequence comparison between barley and wheat miR1126 revealed differences in two nucleotide positions. We confirmed the presence of the mature miR1126 and its corresponding precursor molecule in all developmental stages analyzed (Figure 7F).

### Is miRNA1120, located in the 3′ UTR of a putative protein encoding gene, a functional microRNA?

**Figure 8:**
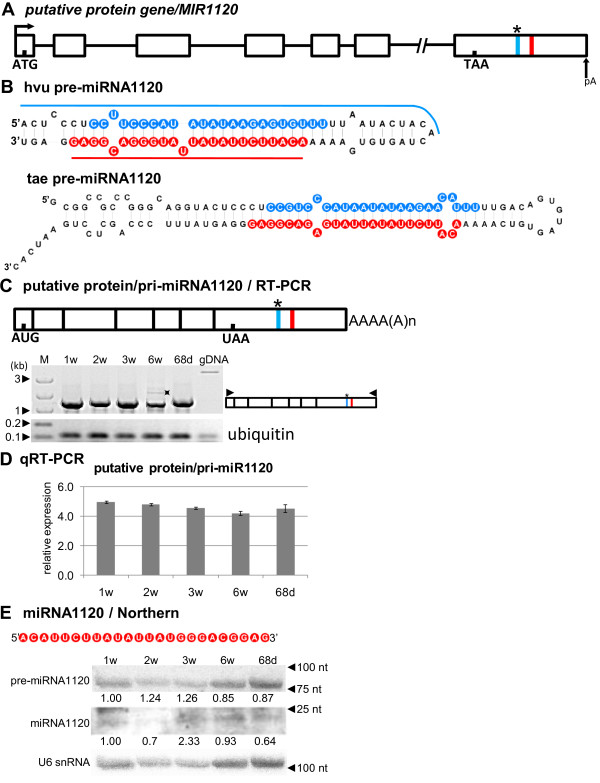
**Schematic representation of the *MIR1120 *gene and its precursor. Detection of pri-, pre- and mature miR1120.** (**A**) *MIR1120* gene structure; black squares in the gene and pri-miRNA1120 schemes show position of the ORF. (**B**) pre-miRNA1120 hairpin structure (ΔG=−42.3 kcal/mol) and its wheat orthologue (ΔG=−63.5 kcal/mol); blue and red lines indicate hybridization regions as described in Figure 1. (**C**) pri-miRNA1120 structure and RT-PCR expression analysis in the five barley developmental stages studied. (**D**) Real-time PCR measurements of total pri-miRNA1120 expression levels; bars on a chart represent standard deviation. Values are shown as the mean ± SD (n=3) from three independent experiments. (**E**) Nucleotide sequence of the mature miR1120 molecule, and detection of pre-miRNA and mature miR1120 using Northern hybridization. U6 was used as a loading control. The level of pre-miRNAs and miRNA was calculated as described in Figure 1. Colors, abbreviations, and symbols as in Figure 1. Asterisk on agarose gel indicates unspecific product.

miRNA1120 was located in the 3^′^ UTR of a protein coding gene. This gene structure is presented in Figure 8A. Interestingly, two introns in the putative protein/*MIR*1120 gene (introns 3 and 5) carry the signatures of U12-type introns, with GU-AG dinucleotides at the 5^′^ and 3^′^ intron ends, and the classic U12 branch point site (UUUCCUCAA) [66,67]. U12 introns are rarely present in plant genes, and it is very rare to have two U12 introns within a single gene [67,68]. The other introns are of the canonical U2-type. Sequences corresponding to the mature miR1120 and miR1120* were located downstream of the stop codon in the 3^′^ UTR (Table 1). The ORF is 1143 base pairs (bp), and encodes a hypothetical protein of 381 aa residues with substantial similarity to a hypothetical rice protein [GeneBank: EEC73666.1].

The sequence and structure of the barley pre-miR1120 precursor show high similarity to its only known wheat orthologue pre-miR1120 (Figure 8B). For mRNA/pri-miR1120 transcripts, we were able to detect only the fully spliced RNA, which is probably due to rapid and efficient splicing of all introns from the primary transcript (Figure 8C). The expression level of the mRNA/pri-miR1120 was almost equal in all developmental stages tested (Figure 8D). However, the level of the mature miR1120 varies during development with the lowest amount in 2-week- and 68-day-old plants (Figure 8E). This suggests the presence of the posttranscriptional mechanisms regulating the miR1120 biogenesis. The pre-miR1120 detected by Northern blot is about 80 nt long and might correspond to the stem-loop structure predicted for pre-miR1120.

Unexpectedly, we found an 85 nt long region which included miR1120/miR1120* and displayed almost 80% sequence similarity to the short transposon element DNA/TcMar-Stowaway [69]. Bioinformatic analysis revealed that this DNA transposable element is overrepresented in the barley genome and exists in around 400 copies. The DNA/TcMar-Stowaway transposon is widespread among both monocot and dicot plants [70,71]. Our finding raises the question whether miR1120 is a true miRNA molecule or it represents a small noncoding RNA such as a siRNA, especially considering its size of 24 nucleotides.

## Conclusions

In this study, we provide experimental evidence for selected mature miRNAs and their pre- and pri-miRNA structures in barley. Seven of the eight analyzed miRNA genes contain one or more introns, and their transcripts are the subject of complex processing events before hairpin pre-miRNA species are diced out from their pri-miRNA precursors. Complex alternative splicing of intron-containing transcripts generated various splice isoforms, which show variations in expression level when studied across five stages of barley growth. Two interesting examples of miRNAs encoded within introns of noncoding genes were identified. The observed tendency to maintain the intron encoding miR156g within the transcript, and preferences in splicing the miR1126-harboring intron, may suggest the existence of special regulation of the levels of intron-derived miRNAs in barley.

The discovery of developmental regulation at the level of expression of mature miRNA species as well as their precursors could help explain the regulatory role of miRNAs in economically important traits of the barley plant.

## Methods

### Plant material and growth conditions

Spring barley seeds, cultivar Rolap [72] were obtained from the Institute of Plant Genetics of the Polish Academy of Sciences (Poznan, Poland). Plants were grown in a greenhouse between August and October of 2009 with seasonal photoperiod and light conditions. Plants were grown in 5 liter pots in medium composed of 2/3 Klasmann TS1 substrate (Klasmann-Deilmann GmbH, Geeste, Germany) and 1/3 sand, and were watered to maintain optimal growth conditions. Whole plants from five growth stages and three biological replicates for each growth stadium were used in experiments. Zadoks decimal code was used to identify the developmental stages [73]. Plants were collected when the first leaf developed, code 11 of Zadoks system (1-week-old plants, Additional file 2: Figure S1 A); after the third leaf developed, code 13 (2-week-old plants, Additional file 2: Figure S1 B); at the beginning of tillering, code 20–21 (3-week-old plants, Additional file 2: Figure S1 C); during stem elongation, code 32–36 (6-week-old plants, Additional file 2: Figure S1 D); when kernels reached milk ripeness, code 75–77 (68-day-old plants, Additional file 2: Figure S1 E). Ten plants from every growth stadium were pooled together and treated as one biological replicate.

### DNA and RNA isolation

Genomic DNA (gDNA) was isolated from 1 g of 6-week-old barley plant tissue using DNeasy Plant Maxi Kit (Qiagen, Hilden, Germany); the concentration and quality of the gDNA were evaluated using a NanoDrop ND-1000 spectrophotometer (NanoDrop Technologies, Wilmington, DE, USA) and confirmed on a 0.6% agarose/EtBr gel. 100 mg of tissue from whole plants, collected 1, 2, 3, and 6 weeks, and 68 days after sowing, was used for total RNA isolation, using a modified method that allows for enrichment of small RNAs [74]. The RNA for Northern blot analyses was extracted twice with 38% phenol solution saturated with 0.1 M sodium acetate (Roti Aqua Phenol, Roth, Karlsruhe, Germany), supplemented with 0.8 M guanidine thiocyanate, 0.4 M ammonium thiocyanate, 0.1 M sodium acetate, 5% glycerol, 0.5% sodium lauroylsarcosine, and 5 mM EDTA. To remove polysaccharides, the Ambion Plant RNA Isolation Aid (Life Technologies, Carlsbad, CA, USA) was used during phenol extraction. Next, three phenol/chloroform and two chloroform extractions were performed. RNA was precipitated in the presence of glycogen using 1.25 vol. of ethanol and 0.5 vol. of 0.8 M sodium citrate in a 1.2 M sodium chloride solution. The quality and quantity of RNA were measured with a NanoDrop ND-1000 spectrophotometer and an Infinite M200 PRO multimode reader (Tecan), RNA integrity was estimated on agarose gels. RNA for RT-PCRs was isolated as described above except for the additional phenol/chloroform and chloroform extractions, which were omitted, and precipitation was achieved with one vol. of isopropanol. DNA contaminants from these samples were removed with RQ1 RNase-free DNase (Promega, Madison, WI, USA). To prove the purity of RNA samples depleted of DNA traces, PCR reactions (thermal profile detailed in “Full-length cDNA of pri-miRNAs amplification”) with 1 μg of DNase-treated RNA as templates and primers amplifying the *MIR171* gene fragment were performed for all biological replicates. In a positive control reaction, 1 ng of gDNA was used (Additional file 3: Figure S2 A).

### Northern blot analysis of pre-miRNAs and mature miRNAs

Denaturing 8 M urea PAGE (15%) was used to separate 30 μg of RNA; the electrophoresis was run in 1x TBE buffer at a temperature of about 55°C. Both the Low Range GeneRuler DNA Ladder (Thermo Scientific, Lithuania) and 10bp DNA ladder (Invitrogen, Carlsbad, CA, USA) were loaded as length markers. RNA was transferred with the aid of a Trans-Blot Electrophoretic Transfer Cell (Bio-Rad) onto Amersham Hybond-NX nitrocellulose (GE Healthcare, Little Chalfont, Buckinghamshire, UK) and fixed using CL-1000 Ultraviolet Crosslinker (UVP). A 1-h pre-hybridization and a 16-h hybridization were performed in hybridization buffer (3.5% SDS, 0.375 M sodium phosphate dibasic, 0.125 M sodium phosphate monobasic) at 50°C for pre-miRNA analysis and at 42°C for miRNAs, with γ^32^P ATP-labeled (6000Ci/mmol; NEN-PerkinElmer, Boston, MA, USA) DNA oligo probes (Sigma). Pre-miRNAs and their respective mature miRNAs were detected on the same blot; a DNA probe complementary to U6 snRNA was used, and the U6 hybridization signal was taken as a loading control. Excess radioactive probe was washed out with 2x SSC, 0.1% SDS buffer, and the blots were exposed for one week to phosphorimaging screen (Fujifilm) and scanned with Fujifilm FLA5100 reader (Fujifilm Co., Ltd., Tokyo, Japan). Blots were quantified with Multi Gauge V2.2 software.

### pri-miRNA 3′ RACE and 5′ RACE experiments

The 5^′^ and 3^′^ RACE cDNA template synthesis and two-step RACE experiments were conducted with the SMARTer RACE cDNA Amplification Kit (Clontech, Mountain View, CA, USA) according to the manufacturer’s protocol. PCR reactions were carried out using the Advantage 2 PCR Enzyme System (Clontech, Mountain View, CA, USA) in a Veriti thermal cycler (Applied Biosystems). Primers were designed for chosen ESTs [GenBank: BG415888.2, AJ475696.1, BQ760548.1, CA003609.1, CA009309.1, BG300360.1, BU974512.1, BJ486588.1] carrying computationally predicted hairpin structure sequences with conserved miRNAs (397b-3p, 159b, 166n, 168a-5p/168a-3p, 171e, 156g, 1126 and 1120, respectively) [46,47]. The primer sequences are listed in Additional file 4. PCR products were cloned into the pGEM T-Easy vector (Promega, Madison, WI, USA) and sequenced (Genomed S.A., Warsaw, Poland).

### 5′ and 3′ genome walking

Four genome walking gDNA libraries were prepared according to the Genome Walker Universal Kit protocol (Clontech, Mountain View, CA, USA), and the PCR reactions were performed with the Advantage 2 PCR Enzyme System (Clontech, Mountain View, CA, USA). Products cloned into the pGEM T-Easy vector (Promega, Madison, WI, USA) were sequenced and compared to 5^′^ and 3^′^ RACE-obtained cDNA fragments using MAFFT software version 6 online [75]. Intron positions were predicted by comparisons of genomic and cDNA sequences using FSPLICE software, http://linux1.softberry.com[53].

### Full-length cDNA of pri-miRNAs amplification

cDNA templates were synthesized with oligo(dT)_15_ (Novazym, Poland) primer and SuperScript III Reverse Transcriptase (Invitrogen, Carlsbad, CA, USA) using 1 μg of DNase-treated RNA as template. 1-, 2-, 3-, and 6-week and 68-day plant cDNAs were diluted 10–15 times, depending on the reverse transcription reaction efficiency, which was estimated by PCR amplification of a ubiquitin cDNA fragment [GenBank: X04133.1]. The purity of cDNA samples containing no gDNA was controlled by PCR amplification of a barley *phosphate transporter 1* (*HvPht1-1*) [GenBank: AF543197.1] promoter fragment of 977 bp with primers anchored upstream of the first P1BS-like motif [76] (Additional file 3: Figure S2 B). Control of gDNA contamination was carried out for all biological replicates. The pri-miRNA amplifications and cDNA purity control reactions were performed with *Taq* DNA polymerase (Thermo Fisher Scientific, formerly Fermentas, Lithuania) or Expand High Fidelity PCR system (Roche, Mannheim, Germany) and two pri-miRNA specific primers (500 nM each) using the following thermal profile - 1 cycle: denaturation at 94°C/1 min, annealing at 65°C/30 s, elongation at 72°C/2 min; 29 cycles: denaturation at 94°C/30 s, annealing at 63°C/30 s (Δ -0.5°C/cycle), elongation at 72°C/2 min; 10 to 13 cycles, depending on the expression level of the pri-miRNA: denaturation at 94°C/30 s, annealing at 53°C/30 s, elongation 72°C/2 min. To improve amplification, Q-Solution (Qiagen, Hilden, Germany) was added to the RT-PCR mix. Genomic DNA template was used as a positive PCR control. Products of the PCR reactions were visualized with ethidium bromide on 1.2% agarose gels with GeneRuler 100 bp Plus or 1kb Plus DNA Ladders (Thermo Fisher Scientific, formerly Fermentas, Lithuania) as length markers. Primer sequences can be found in Additional file 4. Two additional biological replicates were performed for each pri-miRNA amplification presented in the Results and Discussion section (Additional file 5: Figure S3). RT-PCRs were only performed for the qualitative visualization of pri-miRNA processing products.

### Quantitative real-time PCR

Three μg of DNA-free RNA was reverse-transcribed with SuperScript III Reverse Transcriptase (Invitrogen, Carlsbad, CA, USA) and oligo(dT)_15_ (Novazym, Poland) primer. cDNA samples were diluted 2-times and 1μl was used as a template. qPCR was performed with Power SYBR® Green PCR Master MIX (Applied Biosystems, Warrington, UK) and two pri-miRNA-specific primers (final concentration 200 nM each) on 7900HT Fast Real-Time PCR System (Applied Biosystems) in 10 μl reaction volumes in 384-well plates. To amplify the pri-miR168a-5p/miR168a-3p precursor, a reverse primer complementary to exon-exon junction was used due to lack of specific product amplification using the other primers. The following thermal profile parameters were used: 10 min at 90°C, 45 cycles (or 40 cycles for pri-miRNA159b, pri-miRNA166n, pri-miRNA1126 and pri-miRNA1120) of 15 s at 95°C, and 1 min at 60°C. Each real-time PCR reaction was performed independently for three biological replicates, and for every biological replicate three (splicing isoforms analysis) or two (pri-miRNA abundance analysis) technical replicates were performed. The barley *ADP-ribosylation factor 1-like* [GenBank: AJ508228.2] gene fragment of 61 nt was simultaneously amplified and detected as an internal reference [77]. Expression levels were calculated with the relative quantification method (2^-ΔCt^) as fold-change value and presented in a form of log_10_2^-ΔCt^. The R^2^ values of analyzed data (≥0.997) were calculated with LinRegPCR software [78]. Since the pri-miRNAs expression levels are lower than the reference gene, we have shown the expression profiles in a positive data range without changing the actual values by shifting the zero value of the graph’s y-axis to the basal expression level of the whole experiment [79]. qRT-PCR primers were designed and used for pri-miRNA expression level validation. Primers designed and used for the validation of splice isoform levels were complementary to the exon-intron or exon-exon junctions. Primers are listed in Additional file 4.

### Bioinformatics techniques

The sequences of barley miRNA genes - 156g, 159b, 166n, 168a-5p/168a-3p, 171e, 397b-3p, 1120 and 1126 - were deposited in GeneBank [52] [GeneBank: JX121292, JX195499, JX195500, JX195501, JX195502, JX195498, JX195503 and JX195504, respectively]. Sequence analysis was performed with MAFFT version 6, http://mafft.cbrc.jp/alignment/server/index.html[75] and NCBI Blast software, http://blast.ncbi.nlm.nih.gov/Blast.cgi[80]. Secondary structures of pre-miRNAs were predicted using Folder Version 1.11 BETA software with RNAfold, Version 1.6.3 algorithm, http://www.tbi.univie.ac.at/RNA/[81,82]. A pri-miRNA fragment covering at least 120 nt downstream and upstream of miRNA was used to determine miRNA/miRNA* pairing stability. Structures with the lowest minimal folding free energy (ΔG kcal/mol) were shown in this paper. Target prediction was performed with online tools available at http://plantgrn.noble.org/psRNATarget/[83], and *Hordeum vulgare* DFCI gene index HVGI release 11 database http://compbio.dfci.harvard.edu/[84] was used as a software input. Default parameter settings were used.

## Competing interests

The authors declare no competing interests.

## Authors’ contributions

KK, AP and ASB contributed by performing experiments, analyses, creating all the figures and writing the manuscript. AS and EK contributed by performing the RACE and GW experiments. IS contributed by preparing additional material and drafting the manuscript. WK participated in bioinformatics analyses, discussion of the work and assisted in drafting the manuscript. AJ contributed to the experimental analyses, discussion of the work and assisted in drafting the manuscript. ZSK conceived of the study, participated in its design and coordination, participated in the manuscript writing and figures designing. All authors read and approved the final manuscript.

## Supplementary Material

Additional file 1: Table S1 Gene structure and composition of fully spliced and alternatively spliced variants of *MIR156g* and *MIR1126* gene transcripts. The longest ORF detected in each variant is defined by the length of the encoding sequence, position of the ORF within the sequence, numbers of amino acids, and the lowest E-value in blastp analysis. The lengths of the spliced forms are given for the 5^′^ and 3^′^ RACE sequence results, and the lengths of the PCR products obtained using peripheral primers.^1^ Predicted sequence^′^; e - exon; i - intron, nss - no significant similarity in blastp outcome. Click here for file

Additional file 2: Figure S1 Growth stages studied in barley, cultivar Rolap. (A) 1-week-old plants. (B) 2-week-old plants. (C) 3-week-old plants. (D) 6-week-old plants. (E) 68-day-old plants. Click here for file

Additional file 3: Figure S2 The purity of RNA and cDNA samples depleted of gDNA in three biological replicates. (A) RNA samples after DNase treatment. The purity of RNA samples depleted of DNA traces was controlled by PCR amplification of the barley *MIR171* gene. In a positive control reaction, 1 ng of gDNA was used. (B) cDNA samples. The purity of cDNA samples containing no gDNA was controlled by PCR amplification of the barley *phosphate transporter 1* (*HvPht1-1*) promoter fragment of 977 bp long. In a positive control reaction, 1 ng of gDNA was used. 1w: 1-week-old seedlings, 2w: 2-week-old seedlings, 3w: 3-week-old plants, 6w: 6-week-old plants, 68d: 68-day-old plants, gDNA: genomic DNA, H: no template, M: GeneRuler 100 bp Plus DNA ladder. Click here for file

Additional file 4: Table S2 List of primers and hybridization probes used in the experiments. Click here for file

Additional file 5: Figure S3 RT-PCR detection of *MIR* genes transcripts studied in two additional biological replicates (B and C). 1w: 1-week-old seedlings, 2w: 2-week-old seedlings, 3w: 3-week-old plants, 6w: 6-week-old plants, 68d: 68-day-old plants, gDNA: genomic DNA, H: no template control, M: GeneRuler 100 bp Plus or 1kb Plus DNA Ladder. Click here for file
