# Supplementary material for: Developmentally regulated expression and complex processing of barley pri-microRNAs
Source: BMC Genomics. 2013 Jan 16;14:34. doi: 10.1186/1471-2164-14-34 (PMC3558349; doi:10.1186/1471-2164-14-34)
Supplement: Additional file 1: Table S1 — Gene structure and composition of fully spliced and alternatively spliced variants of MIR156g and MIR1126 gene transcripts. The longest ORF detected in each variant is defined by the length of the encoding sequence, position of the ORF within the sequence, numbers of amino acids, and the lowest E-value in blastp analysis. The lengths of the spliced forms are given for the 5′ and 3′ RACE sequence results, and the lengths of the PCR products obtained using peripheral primers.1 Predicted sequence′; e - exon; i - intron, nss - no significant similarity in blastp outcome. [file 1471-2164-14-34-S1.pdf]

Table S1 Gene structure and composition of fully spliced and alternatively spliced variants of *MIR156g*and *MIR1126*genes. The longest ORF detected in each variant is described by the length of the encoding sequence, position of the ORF within the sequence, amino acids number and the lowest E-value in blastp analysis. The length of the spliced forms is given for the 5' and 3' RACE sequence results and the length of PCR products obtained using peripheral primers.[1]predicted sequence; e - exon; i - intron, nss - no significant similarity in blastp outcome.

**Splicing variant**	**Length [bp]**	**The longest ORF [bp], position**	**Amino acids**	**Blastp, E-value**
***MIR156* gene**	9759	441, (1388-1828)	146	5.7
Fully spliced form:<br>e1,e2,e3,e4(83bp),e5(145bp),e6(513bp)[1]	1051	285, (115-399)	94	0.47
**I**: e1,i1,e2,i2(990bp)	2083/2079	441, (1388-1828)	146	5.7
**II**: e1,i1,e2,e3,e6	1674/1484	369, (957-1325)	122	1.1
**III**: e1,i1,e2,e3,e5(84bp),e6(323bp)	1568	453, (957-1409)	150	3.0
**IV**: e1,i1,e2,e3,e4(79bp),e5(145bp),e6(190bp)	1575	324, (312-635)	107	1.1
**V**: e1,i1,e2,e3,e4(83bp),e5(145bp),e6(323bp)	1712	324, (312-635)	107	1.1
**VI**: e1,e2,e3,e4(83bp),e5(145bp),e6(323bp)	861	285, (115-399)	94	0.47
**VII**: e1,e2,e3,e5(87bp),e6(323bp)	720	372, (115-489)	123	2.7
**VIII**: e1,e2,e3,e6(323bp)	633	360, (115-474)	119	1.0
***MIR1126* gene**	3297	276, (349-624)	91	nss
Fully spliced form:e1,e2,e3,e4,e5,e6,e7	783	180, (457-636)	59	3.1
**I**: e1,e2,i2,e3,i3,e4,e5,e6,i6,e7	2451	276, (199-474)	91	nss
**II**: e1,e2,i2,e3,i3,e4,e5,e6,e7	2291	276, (199-474)	91	nss
**III**: e1,i1,e2,e3,e4,e5,e6,i6,e7	1093	180, (607-786)	59	3.1
**IV**: e1,i1,e2,e3,e4,e5,e6,e7	933	180, (607-786)	59	3.1
**V**: e1,e2,e3,e4,e5,e6,e7	783	180, (457-636)	59	3.1
