# Supplementary figures and images for: Developmentally regulated expression and complex processing of barley pri-microRNAs
Source: BMC Genomics. 2013 Jan 16;14:34. doi: 10.1186/1471-2164-14-34 (PMC3558349; doi:10.1186/1471-2164-14-34)

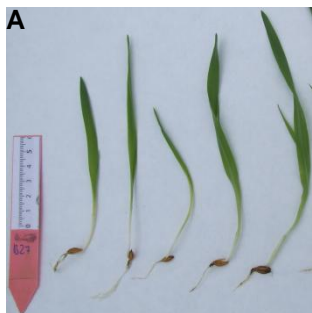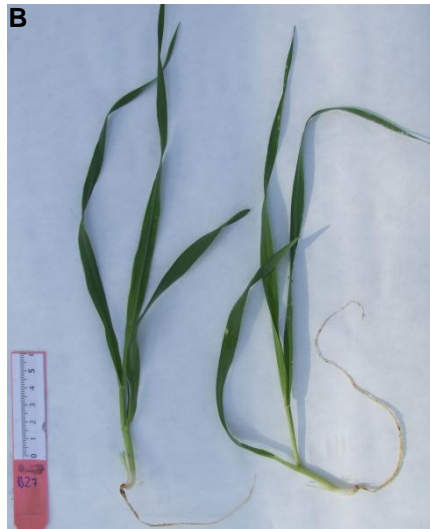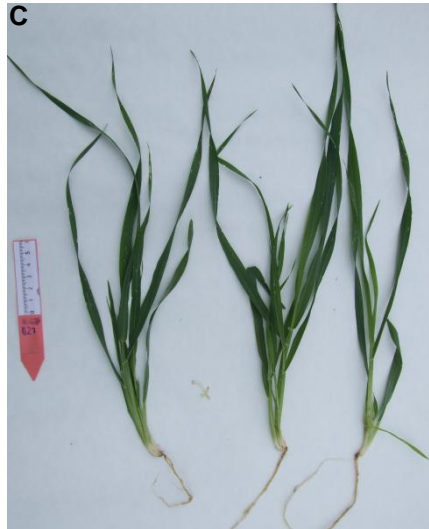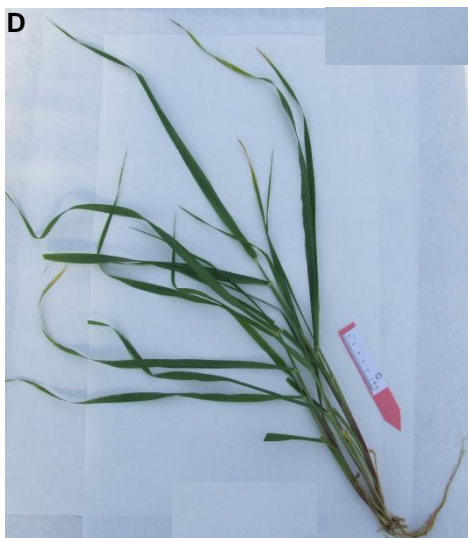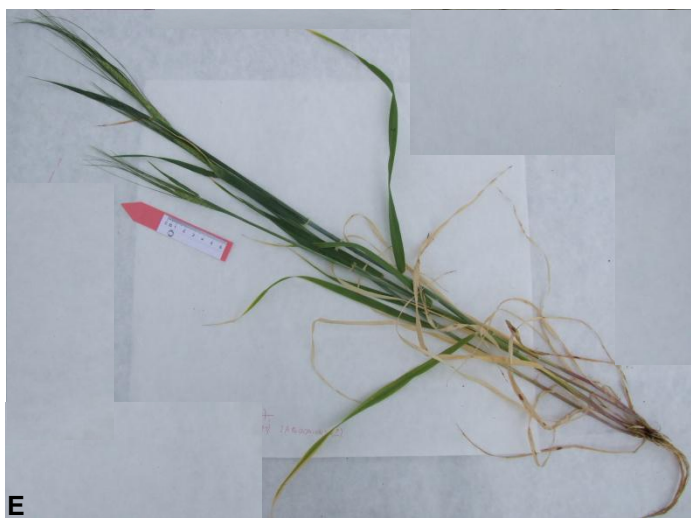

Supplement: Additional file 2: Figure S1 — Growth stages studied in barley, cultivar Rolap. (A) 1-week-old plants. (B) 2-week-old plants. (C) 3-week-old plants. (D) 6-week-old plants. (E) 68-day-old plants. [file 1471-2164-14-34-S2.pdf]

## A RNA samples after DNase treatment

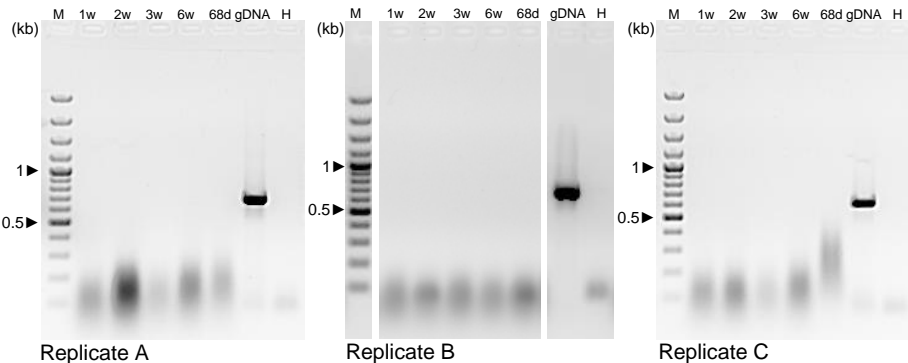

## B cDNA samples

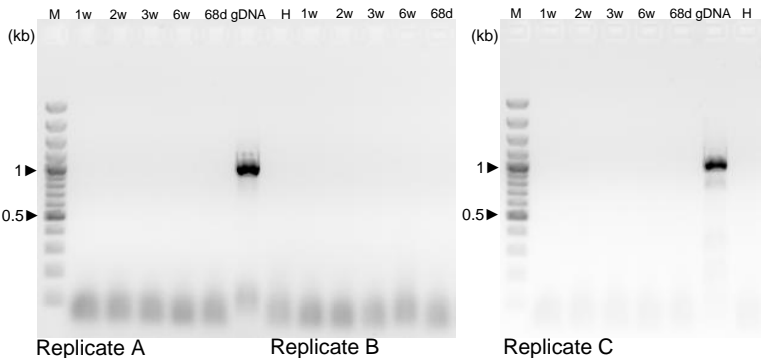

Supplement: Additional file 3: Figure S2 — The purity of RNA and cDNA samples depleted of gDNA in three biological replicates. (A) RNA samples after DNase treatment. The purity of RNA samples depleted of DNA traces was controlled by PCR amplification of the barley MIR171 gene. In a positive control reaction, 1 ng of gDNA was used. (B) cDNA samples. The purity of cDNA samples containing no gDNA was controlled by PCR amplification of the barley phosphate transporter 1 (HvPht1-1) promoter fragment of 977 bp long. In a positive control reaction, 1 ng of gDNA was used. 1w: 1-week-old seedlings, 2w: 2-week-old seedlings, 3w: 3-week-old plants, 6w: 6-week-old plants, 68d: 68-day-old plants, gDNA: genomic DNA, H: no template, M: GeneRuler 100 bp Plus DNA ladder. [file 1471-2164-14-34-S3.pdf]

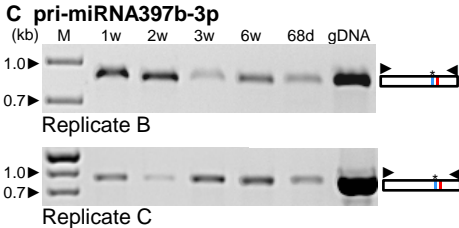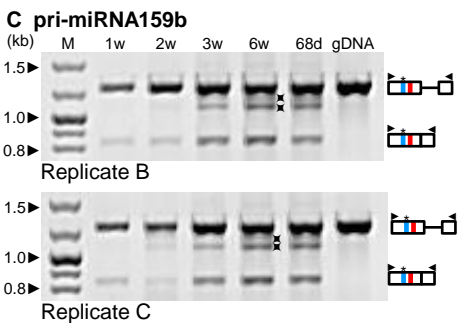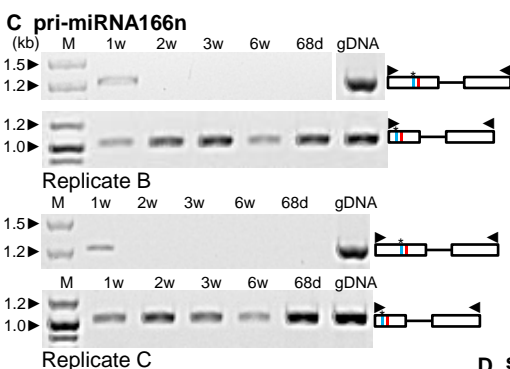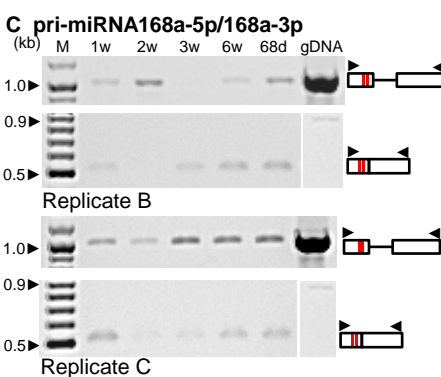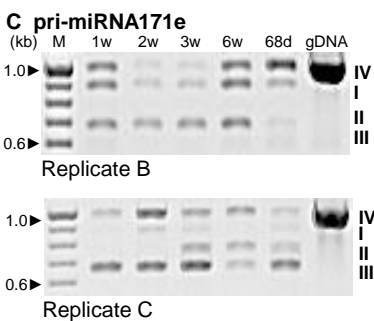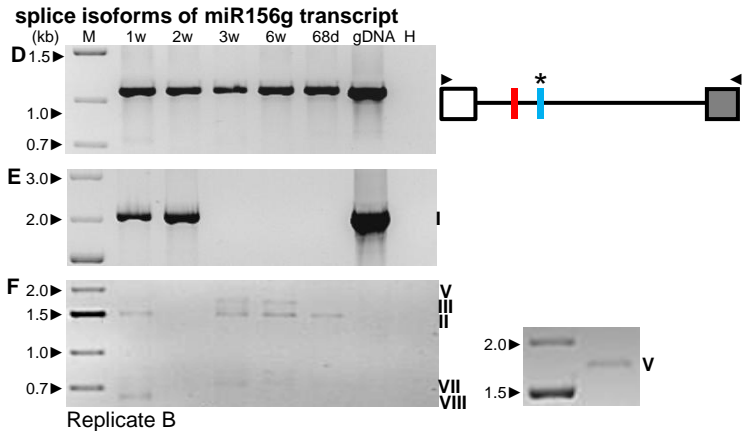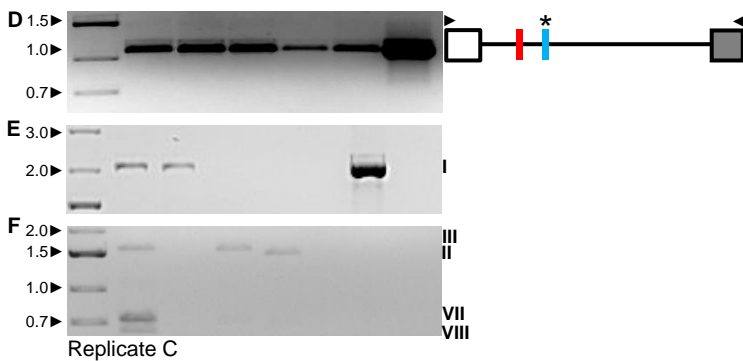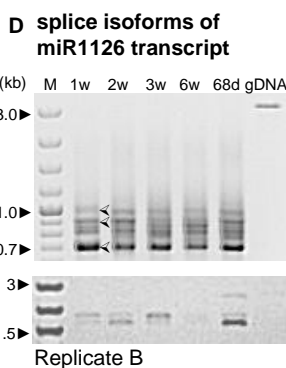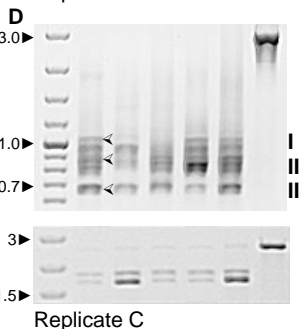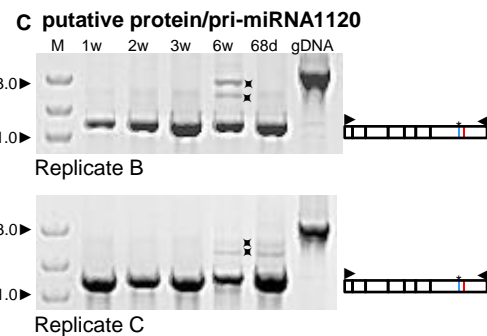

Supplement: Additional file 5: Figure S3 — RT-PCR detection of MIR genes transcripts studied in two additional biological replicates (B and C). 1w: 1-week-old seedlings, 2w: 2-week-old seedlings, 3w: 3-week-old plants, 6w: 6-week-old plants, 68d: 68-day-old plants, gDNA: genomic DNA, H: no template control, M: GeneRuler 100 bp Plus or 1kb Plus DNA Ladder. [file 1471-2164-14-34-S5.pdf]
