# Supplementary material for: Developmentally regulated expression and complex processing of barley pri-microRNAs
Source: BMC Genomics. 2013 Jan 16;14:34. doi: 10.1186/1471-2164-14-34 (PMC3558349; doi:10.1186/1471-2164-14-34)
Supplement: Additional file 4: Table S2 — List of primers and hybridization probes used in the experiments. [file 1471-2164-14-34-S4.pdf]

Table S2 List of primers and hybridization probes used in experiments

primer	sequence (5’→ 3’)	miRNA	application
ASO141	CACAGCAGACGTTCAACACGCAGA	156g	5’ RACE, 5’ GW
ASO142	CACACCATCATCAACCCTCCACCGT	156g	5’ RACE, 5’ GW
APO342	TCGTTCCCACAGATCTGGGAGG	156g	5’ RACE
APO343	AGCTTTCCTCTTGCAGCCAAGTGTTGT	156g	5’ RACE
APO200	CAAAGCCTCATCCTGGCTCTTCTCG	156g	5’ GW
APO201	TGCATGGATAGACTGATGGCGGAAC	156g	5’ GW
APO255	ACCAGAAAGGGCAACTGGAATTCATGATAG	156g	5’ GW
APO256	GGCACAACTTTCTCCTTTTAATCACAT	156g	5’ GW
APO264	AAAGGAACAACCCACACATAAGCCAAGAAC	156g	5’ GW
APO265	CTAGGGCACAAACCATAGCACAACAAAAGA	156g	5’ GW
APO139	TCTGCGTGTTGAACGTCTGCTGTG	156g	3’ RACE, 3’ GW
APO140	ACGGTGGAGGGTTGATGATGGTGT	156g	3’ RACE, 3’ GW
APO202	CACATGAGCCCTATCTTGYCCGATT	156g	3’ GW
APO203	GGCCTAGCCCCCTGCTACTTG	156g	3’ GW
APO243	ATGCATGGATGCTTAGGTGACGTGTTAT	156g	3’ GW
APO244	TCCCCTGATGCTAAACAAAATCCCTCAAT	156g	3’ GW
APO257	GCGCCATAGTAGGTTCCTCAAGAATTGTGT	156g	3’ GW
APO258	TCAATACCGACTCCAGTGATAGGGTCACAT	156g	3’ GW
APO268	CTGCTGTAGGATTGCATACTTTTGCTTCGT	156g	3’ GW
APO269	TATTGTTGCAAGAGGCAACTTTGTTGACAT	156g	3’ GW
ASO17	ATGCTACCTTCCAGGAGATGAAGTGAC	156g	3’ GW
ASO18	CTACTTTCAGGTGTGCCTTTTGTAGC	156g	3’ GW
APO279	GTGGTATACAACCAGATCGAG	156g	RT-PCR
APO280	TACCAACAGATATTGGTACTCA	156g	RT-PCR
APO281	TGCTATACTGAGTTTTTTATTTCGT	156g	RT-PCR
APO300	GAATAGAGAGATGGTCATTATGG	156g	RT-PCR
APO341	CGTTCCCACAGATCTGGGAG	156g	RT-PCR
HvReTi14_F	CTGCGTCTGATAAAGAGGACG	156g	Real time PCR – pri-miRNA
HvReTi14_R	ATGCATGGATAGACTGATGGC	156g	Real time PCR – pri-miRNA
KK327	GGCGTATGCGTACATGGGT	156g	Real time PCR – with intron
KK328	TGTGTGCTCACTCTCTTCTGTC	156g	Real time PCR – with intron
KK329	GCGTATGCGTACATGGGCAG	156g	Real time PCR – without intron
KK330	CCTCTTGCAGCCAAGTGTTGTAG	156g	Real time PCR – without intron
APO131	GTGCTCACTCTCTTCTGTC	156	probe - mature miRNA

KK193	GCTGACAGAGAGAGGGTGAGCACGCAACAGCAGCTGC GTCTATCT	156g	probe - precursor
KK3	ACGCAAGAAAGAGCTTGCACCCAGA	159b	5’ RACE
KK4	CTGGACCGTGGAGGAGAAAAATCTGC	159b	5’ RACE
KK2	TCGCTGACCGCTGTTTGGATTGAAGG	159b	3’ RACE, 3’ GW
KK5	GGCTTGTGGTTTGCATGATCGAGGA	159b	3’ RACE
KK22	ACGCAAGAAACAGCTTGCACCCAGA	159b	5’ GW
KK30	GCATCAATCCGTCCTCTATCAAATCCTGCA	159b	5’ GW
KK29	GGTGTTTTGTTTTCCCCTCAAGGTAATGA	159b	3’ GW
KK38	ACACATCAACAGCATCACAGAATAGT	159b	RT-PCR
KK39	TGCTCAAACAGAGACTGATTACATACTC	159b	RT-PCR
HvReTi_4_F	TGTTTTGGATTTGGTTTGGAG	159b	Real time PCR – pri-miRNA
HvReTi_4_R	ACCACAAGCCTATCTCCTCGT	159b	Real time PCR – pri-miRNA
KK313	GATCTCTCCTTGAGCTTGAACT	159b	Real time PCR – with/without intron
KK314	TCCGTCCTCTATCAAATCCTTG	159b	Real time PCR – without intron
KK315	CAAAGTACAAAGCATAGCCTGA	159b	Real time PCR – with intron
KK65	CAGAGCTCCCTTCAATCCAAA	159	probe - mature miRNA
KK188	TGCACAAACCCTTCCGGTAGACCCTTCATTGGAATGAT AGGAGCTC	159b	probe - precursor
KK8	TAGCTGCAGGCAGGCATGGGGAGTA	166n	5’ RACE, 5’ GW
KK9	CACATGGACAGAGGCACAGGTGGAG	166n	5’ RACE, 5’ GW
KK6	GCGTCATCTTCTTCGTTTCGCGTCA	166n	3’ RACE, 3’ GW
KK7	TCGTTTCGCGTCATGGTTGTCGAG	166n	3’ RACE, 3’ GW
KK40	AGGGCAGGCAGTTCATGGCCTGAC	166n	RT-PCR
KK41	CATAAATGGATTTTCACTTCTTTCC	166n	RT-PCR
KK49	CGTAGCTGGTCTAACCACCACACA	166n	RT-PCR
HvReTi_5_F	CAGCGTCATCTTCTTCGTTTC	166n	Real time PCR – pri-miRNA
HvReTi_5_R	AGGTGGAGCTCACAAGAACAC	166n	Real time PCR – pri-miRNA
KK338	TCCATCATGGTTCAGGTTCAC	166n	Real time PCR – with/without intron
KK339	AGAAAACAGTAGTATCTGGCTGC	166n	Real time PCR – with intron
KK340	CTATTCCAATCATCATCAGCCTTC	166n	Real time PCR – without intron
KK66	GGGGAATGAAGCCTGGTCCGA	166	probe - mature miRNA, precursor L
KK189	CGAGCGTCTCTCTTTCGGACCCGGCGTCATTCCCCTCGA	166n	probe – precursor

	CAACCAT		S
KK12	CAGGTGCCAGAGCACCGACCAATTC	168a-5p/168a-3p	5’ RACE, 5’ GW
KK13	GAGGGAGCGAAAGCGGTGGTGGT	168a-5p/168a-3p	5’ RACE, 5’ GW
KK10	CCCCTCCCCACCCGCAAAGTCCA	168a-5p/168a-3p	3’ RACE, 3’ GW
KK11	CCTCGGGCTCGCTTGGTGCAGAT	168a-5p/168a-3p	3’ RACE, 3’ GW
KK42	ACGCAGCGACCAGTCGCCGTAGGTA	168a-5p/168a-3p	RT-PCR
KK44	GAATCAAAAGGCTGAAATTCTGCCA	168a-5p/168a-3p	RT-PCR
HvReTi_19F	GCCTTGCACCAAGTGAATC	168a-5p/168a-3p	Real time PCR – pri-miRNA
HvReTi_6_R	TACAACCTGGAACGAATCCAA	168a-5p/168a-3p	Real time PCR – pri-miRNA
KK316	CGCCCTCCCGAATTGGAT	168a-5p/168a-3p	Real time PCR – without intron
KK317	CTCGTAGCATCCCATACAGCAC	168a-5p/168a-3p	Real time PCR – with/without intron
KK318	TGCCTGACCGATCTAATCCTCT	168a-5p/168a-3p	Real time PCR – with intron
KK67	GTCCCGATCTGCACCAAGCGA	168	probe - mature miRNA
KK190	GCTCCGATTCACTTGGTGCAAGGCGGGATCCGGCCCGTCGG	168a-5p/168a-3p	probe – prekursor, 168a-5p
KK24	TGCAACGTGGGTGATGGCGCGTGTACGC	171e	5’ RACE, 5’ GW
KK37	GGAGGAGCTAAGCTAGGTATGGCGACCG	171e	5’ RACE
KK14	CCACTCCATCGCCTCCCCTCTCTTC	171e	3’ RACE, 3’ GW
KK15	CCGTCTCTCCTCCTTGCGGGTTGAT	171e	3’ RACE, 3’ GW
KK16	ACCTCCATACCTGCAACGTGGGTGA	171e	5’ GW
KK45	ATCGCCTCCCCTCTCTTCTACCTC	171e	RT-PCR, RNA purity control
KK60	TGGTAGTTAGCCTGCCTGTAGATC	171e	RNA purity control
KK200	ATGAAGGCGTGGGGATTACAC	171e	RT-PCR
HvReTi_12_F	CTCTCCTCCTTGCGGGTTGAT	171e	Real time PCR – pri-miRNA
HvReTi_18R	GCGGAGGAGCTAAGCTAGGTA	171e	Real time PCR – pri-miRNA
KK319	CATCACCCACGTTGCAGGTA	171e	Real time PCR – II/IV
KK320	TCACACACACAAGGCAAACCTG	171e	Real time PCR – IV
KK325	ATCACCCACGTTGCAGGCAG	171e	Real time PCR – without III
KK326	ACTGCAACCATCTGAAGGGAGG	171e	Real time PCR – without III
KK335	ACGCATGTGTTCGTCACTTCGTC	171e	Real time PCR –

			I
KK336	ACACACACAAGGCAAACCTGCA	171e	Real time PCR – I
KK337	TGGTAGTTAGCCTGCCTGG	171e	Real time PCR – II
KK68	GATATTGGCACGGCTCAATCA	171	probe - mature miRNA, precursor S
KK191	GCTCCCTCCGGCGTGGTCTGAGTGAGTCGAGCCAACAT CATAG	171e	probe – precursor L
KK27	CCATGATTCCTGCTTGCGTCTACGG	397b-3p	5’ RACE, 5’ GW
KK28	TACTGGGCAAGACGAAGAGGAGGAAGA	397b-3p	5’ RACE, 5’ GW
KK25	TCGGCACCACATGAGCCTACAGAAGTA	397b-3p	3’ RACE, 3’ GW
KK26	AAGAGGAACGGAACGGAAGGCTGATT	397b-3p	3’ RACE, 3’ GW
KK47	ACGAGACAACAGCGGCTCGCCACT	397b-3p	RT-PCR
KK55	ATTTGAGATTCAGATGATTACTCAAGC	397b-3p	RT-PCR
HvReTi_16F	TCATCAACGCTGCACTCAAC	397b-3p	Real time PCR – pri-miRNA
HvReTi_16R	ATCTCCCGGTTTCAAAAGCTA	397b-3p	Real time PCR – pri-miRNA
KK69	CCGTTGAGTGCAGCGTTGATG	397	probe - mature miRNA
KK192	CACCGAGGCCGGAGCGGTTCACCGGCGCTGCACGCAA TGACGCCT	397b-3p	probe - precursor
KK226	TCACCGGCGCTGCACGCAATG	397b-5p	probe - 397b-5p
KK80	ATGCTCCTATCTAACTCCTCCGTCCC	1120	5’ RACE, 5’ GW
KK100	TCCCATGACACTGCAGGAATCAGACCAA	1120	5’ RACE, 5’ GW
KK77	ATTTCGTGAGCCTGGAGCTGCTTGT	1120	3’ RACE
KK78	TTGGTCTGATTCCTGCAGTGTCACGGG	1120	3’ RACE
KK159	ATGCGAATGACCTGACTGTATC	1120	3’ GW
KK126	GTACGAAAGACTGTACTGCAAC	1120	3’ GW
KK125	CCATGAAACCCTAAGACCACCGT	1120	RT-PCR
KK173	TCAACGGCAATGTTCCTCTGCT	1120	RT-PCR
HvReTi_1_F	CTGCAGTGTCACGGGACTTAT	1120	Real time PCR – pri-miRNA
HvReTi_1_R	ATGCACCCAAATATGCTCCTA	1120	Real time PCR – pri-miRNA
KK180	CTCCGTCCCATAATATAAGAATGT	1120	probe - mature miRNA
KK186	TGTAGTATTAAAAACACTCTTATATATGGGAAGGAGGG AGTAG	1120	probe - precursor
KK89	TGTTTTGTGCTGAGTTAGGTGGAAGGT	1126	5’ RACE
KK90	GTTCCTTTGTTCTACTCCCTCTGTTCCT	1126	5’ RACE
KK91	TATTTAGGGTCGGTAAGCATCCATACA	1126	3’ RACE
KK92	ACATTAATAAATACTCCCTCCGTTCCTA	1126	3’ RACE

KK157	GGAACATTTATGTAGGTGAGGTCGTCTCG	1126	5’ GW
KK158	GTGAGGTCGTCTCGACCTTCCACCTAACTCAGC	1126	5’ GW
KK116	AGAAAGGTGCCAACAGTGCATTGCCGT	1126	3’ GW
KK117	TGGTTTGTTCAAATCGACAGTCCGACGCTC	1126	3’ GW
KK108	ACTTATGTTCCCACTGAATCGAC	1126	RT-PCR
KK167	TCGAGTGAACTCACATGAGCTGA	1126	RT-PCR
HvReTi_25F	GGAACAAAATGAGTGAACTTACA	1126	Real time PCR – pri-miRNA
HvReTi_2R	TTAGGGTCGGTAAGCATCCAT	1126	Real time PCR – pri-miRNA
APO400	CAACAGATATTTTCTTGCTATTGC	1126	Real time PCR – with intron
APO401	GAGCGACAACATGCAAACTGA	1126	Real time PCR – with/without intron
APO403	TTGAGGGAATTTATGGAAGGCA	1126	Real time PCR – without intron
KK182	TTCCGTATGTAGTCCATAGTTGA	1126	probe - mature miRNA
KK187	TTTTTTTAGAGATTTCCCTATGGGCTACATATGGATATA	1126	probe - precursor
APO395	AATAACGTGTTTTGGGCAAACTT	HvPht1-1	cDNA purity control
APO396	AAGGGACATTTCCTCGCTACTTG	HvPht1-1	cDNA purity control
APO387	CGTGACGCTGTGTTGCTTGT	ADP	Real time PCR
APO388	CCGCATTCATCGCATTAGG	ADP	Real-time-PCR
UBQF	CCTGCGTGGTGGCAAGTAAG	UBQ	RT-PCR
UBQR	ACAACCAGACATGCTCCAACCT	UBQ [85]	RT-PCR
U6	TCATCCTTGCGCAGGGGCCA	U6	probe
